# AHNAK enables mammary carcinoma cells to produce extracellular vesicles that increase neighboring fibroblast cell motility

**DOI:** 10.18632/oncotarget.10307

**Published:** 2016-06-27

**Authors:** Thaiomara A. Silva, Basílio Smuczek, Iuri C. Valadão, Luciana M. Dzik, Rebeca P. Iglesia, Mário C. Cruz, André Zelanis, Adriane S. de Siqueira, Solange M.T. Serrano, Gary S. Goldberg, Ruy G. Jaeger, Vanessa M. Freitas

**Affiliations:** ^1^ Department of Cell and Developmental Biology, Institute of Biomedical Sciences (ICB), University of Sao Paulo, Sao Paulo, Brazil; ^2^ Center of Facilities and Support Research, Institute of Biomedical Sciences (ICB), Sao Paulo, Brazil; ^3^ Department of Science and Technology, Institute of Science and Technology, Federal University of Sao Paulo (ICT-UNIFESP), Sao Jose dos Campos, Brazil; ^4^ Special Laboratory of Applied Toxinology, Center of Toxins, Immune-Response and Cell Signaling, Butantan Institute, Sao Paulo, Brazil; ^5^ Department of Molecular Biology, School of Osteopathic Medicine, Rowan University, Stratford, New Jersey, USA

**Keywords:** extracellular vesicles, cancer, microvesicles, AHNAK, cancer associated fibroblasts

## Abstract

Extracellular vesicles play important roles in tumor development. Many components of these structures, including microvesicles and exosomes, have been defined. However, mechanisms by which extracellular vesicles affect tumor progression are not fully understood. Here, we investigated vesicular communication between mammary carcinoma cells and neighboring nontransformed mammary fibroblasts. Nonbiased proteomic analysis found that over 1% of the entire proteome is represented in these vesicles, with the neuroblast differentiation associated protein AHNAK and annexin A2 being the most abundant. In particular, AHNAK was found to be the most prominent component of these vesicles based on peptide number, and appeared necessary for their formation. In addition, we report here that carcinoma cells produce vesicles that promote the migration of recipient fibroblasts. These data suggest that AHNAK enables mammary carcinoma cells to produce and release extracellular vesicles that cause disruption of the stroma by surrounding fibroblasts. This paradigm reveals fundamental mechanisms by which vesicular communication between carcinoma cells and stromal cells can promote cancer progression in the tumor microenvironment.

## INTRODUCTION

Cancer kills over 8 million people every year, working out to a person about every 4 seconds around the world [1]. Breast cancer constitutes the second most frequent cancer worldwide. Breast cancer accounts for about 12% of all new cancer cases, affects about 1 in 8 women, and is responsible for 500 thousand deaths every year [2, 3]. A better understanding of cancer progression is clearly needed to prevent and treat these cancers.

Mammary carcinoma cells interact with the surrounding microenvironment including extracellular matrix (ECM) components and normal stromal cells including fibroblasts [4]. Interactions between these cells create an environment needed to supporting tumor growth, invasion, and metastasis [5, 6]. These interactions can be mediated by direct contact by intercellular junctions or secreted factors that diffuse between cells independently or within extracellular vesicles [7].

Extracellular vesicles have recently emerged as important factors that regulate tumor progression in the microenvironment [7, 8]. These include exosomes that are between 30 to 100 nanometers in diameter, and microvesicles that are in the range of 100 to 1000 nanometers in diameter [9]. These vesicles transmit signals that regulate many aspects of cancer progression including angiogenesis and degradation of the extracellular matrix leading to tumor invasion and metastasis [7, 8, 10].

Extracellular vesicles from different cell types have been characterized by proteomic analysis [11, 12]. For example, exosomes-like vesicles from breast tumor cells (MDA-MB-231 and MCF-7 cells) were found to contain proteins including actin, annexins (A1, A2, A5), pyruvate kinase isoenzymes (M1/M2), tubulin (α and β), heat shock proteins (HSP 90α), histone (H4), and integrin α-2 [13]. In addition, relatively low levels of AHNAK (Neuroblast differentiation-associated protein; desmoyokin) and myoferlin proteins were found in vesicles from MCF-7 cells [13]. The AHNAK protein was also observed in proteomic analysis of isolated vesicles from prostate tumor cells [14].

Here, we investigated the role of AHNAK in extracellular vesicle formation and exchange between mammary carcinoma cells and neighboring fibroblasts. We determined the number, size, composition, and potential function of these vesicles. Interestingly, these vesicles traveled with a directional tendency from aggressive mammary carcinoma cells to fibroblasts. Secretome analysis found high levels of AHNAK in vesicles from aggressive MDA-MB-231 cells. We also found higher expression of AHNAK in human breast tumor samples compared to normal tissue. These data indicate that AHNAK does not promote cell growth, but does enable mammary carcinoma cells to produce extracellular vesicles, and that these vesicles travel to, and increase the migration of, stromal fibroblasts. Thus, AHNAK appears to promote extracellular vesicle production by mammary carcinoma cells in order to increase fibroblast migration with consequent modification of the tumor microenvironment.

## RESULTS

### Mammary carcinoma cells and fibroblasts produce and exchange vesicles with each other

Signaling between tumor cells and stromal fibroblasts is crucial for development of the tumor microenvironment [15]. However, the role of extracellular vesicles in this process is not well understood. We utilized mammary carcinoma cells and fibroblasts from normal mammary tissue as a model to study this form of intercellular communication.

**Figure 1:**
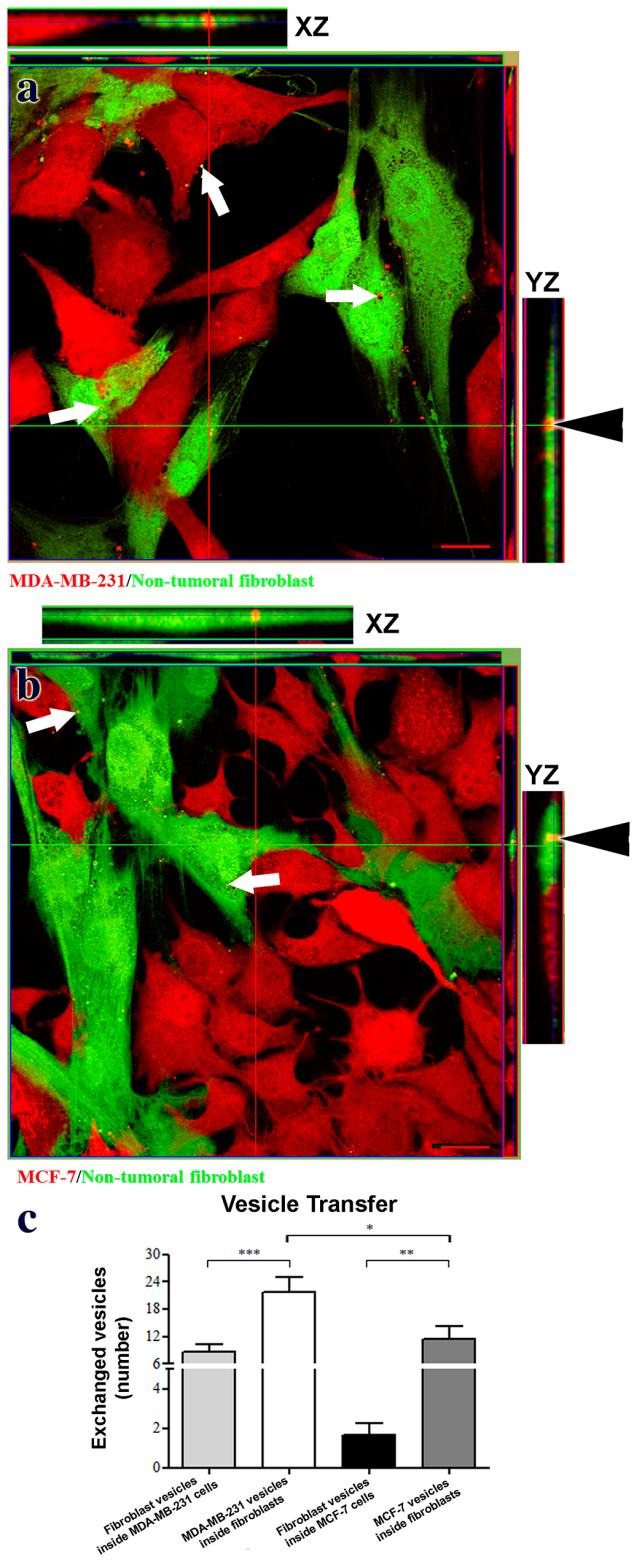
Mammary carcinoma cells exchange vesicles with nontransformed mammary fibroblasts Panels **a.** and **b.** present confocal images showing exchange of vesicular structures between fibroblasts (green) and tumor cells (red, arrows in panels a and b). Orthogonal projections verify the presence of tumor vesicles inside fibroblasts (arrowheads in panels a and b). Panel **c.** presents quantitation of vesicle transfer as numbers of vesicles transferred in a field containing 20 cells (mean+SEM, n≥5). Single, double, and triple asterisks indicate statistically significant differences of p<0.05, p<0.01 and p<0.001, respectively. Scale bars = 20 μm.

We used MDA-MB-231 and MCF-7 cells to represent highly aggressive and less aggressive mammary carcinoma cells, respectively [16]. Nontransformed mammary fibroblasts were expanded from primary cultures derived from normal human breast tissue. Specifically, our objective was to evaluate vesicle exchange dynamics between normal and tumor cells. Each cell type was stained with different dyes to identify components that transferred between tumor cells and nontransformed fibroblasts in co-culture. As shown in Figure 1a and 1b, vesicles can be seen traveling between mammary carcinoma cells and fibroblasts. This vesicular transfer is confirmed by orthogonal projections from confocal optical sections. Quantitation of these data found that vesicles traveled more frequently from tumor cells to fibroblasts than from fibroblasts to tumor cells, as shown in Figure 1c. In addition, about 4 times as many vesicles traveled to fibroblasts from MDA-MB-231 cells than from MCF-7 cells. Thus, more aggressive MDA-MB-231 carcinoma cells transferred more vesicles than less aggressive MCF-7 cells.

### Vesicles and cell membrane protrusions can be visualized by electron microscopy

**Figure 2:**
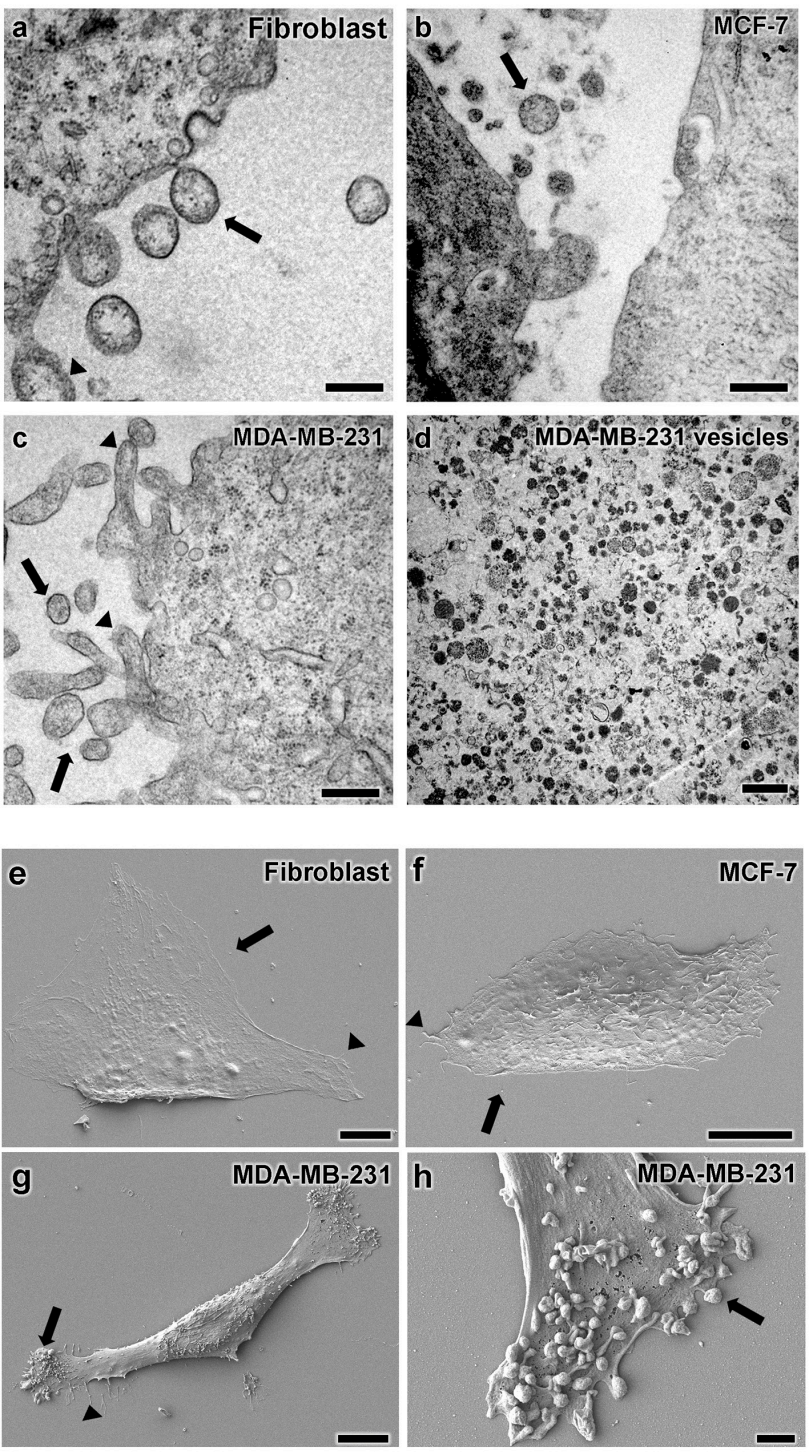
Vesicular structure characterization by electron microscopy Transmission electron microscopy analysis of **a.** fibroblasts, **b.** MCF-7 cells, and **c.** MDA-MB-231 cells are shown with vesicular structures with size ranging from 200-400 nm indicated by arrows. TEM also found membrane protrusions, mostly in MDA-MB-231 cells as indicated by arrowheads in panel c. **d.** Vesicles isolated from MDA-MB-231 cells consisted of a heterogeneous population with different diameters and electron-densities. Scanning electron microscopy images of **e.** fibroblasts, **f.** MCF-7 cells, and **g-h.** MDA-MB-231 cells display cell protrusions indicated by arrowheads and vesicles indicated by arrows, particularly on MDA-MB-231 cells. Average vesicles size is approximately 200 nm in diameter. Scale bars for A-C = 200 nm, D = 500 nm, E-G = 10 μm, and E = 1 μm.

Electron microscopy was used to analyze the morphology of vesicles produced by mammary carcinoma cells and fibroblasts used in this study. Transmission electron microscopy (TEM) revealed cell protrusions that appeared to produce microvesicles (indicated by arrowheads in Figure 2a-2c). In general, more protrusions were found in carcinoma cells than in nontransformed fibroblasts.

In addition to cell protrusions, vesicular structures were also found inside and outside of cells. Most extracellular vesicles had diameters ranging from 200 to 400 nm (Figures 2a-2c). However vesicles isolated from conditioned medium were observed with different electron densities and diameters ranging from 200 to 700 nm as shown in Figure 2d.

Scanning electron microscopy (SEM) was utilized to further examine the morphology of cell protrusions seen by TEM. These images, shown in Figures 2e-2h confirm the potential of cells to form vesicular structures between 200 to 700 nm in diameter. These were particularly prominent in MDA-MB-231 cells (Figure 2h). In general, these data highlight the production of extracellular vesicles compatible in size and shape with microvesicles over 100 nm in diameter.

### Extracellular vesicle size examined by nanoparticle tracking analysis (NTA)

**Figure 3:**
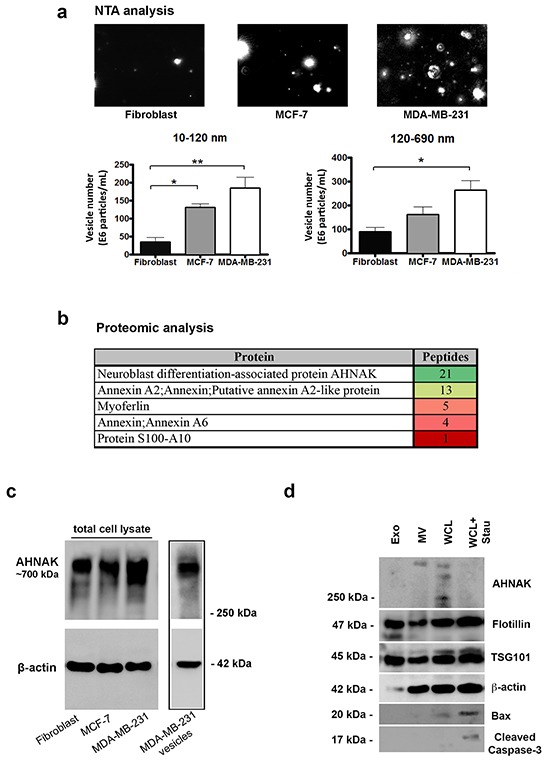
Nanoparticle tracking and LC-MS/MS analysis of extracellular vesicles **a.** NTA images of vesicles isolated from MDA-MB-231 cells, MCF-7 cells, and fibroblasts as indicated. Vesicles were quantitated into sizes between 10-120 nm and 120-690 nm by NanoSight technology. Results are shown as the number of vesicles within each size range (mean+SEM, n=3). Single and double asterisks indicate statistically significant differences of p<0.05 and p<0.01, respectively. Scale bar = 5 μm. **b.** Selected proteins identified in vesicles from MDA-MB-231 cells by trypsin digestion and LC-MS/MS analysis are shown along with their peptide number and relative abundance color coded from green to red, as indicated. **c.** AHNAK and β-actin protein levels in nontransformed fibroblasts, MCF-7 cells, MDA-MB-231 cells, and in isolated vesicles from MDA-MB-231 cells were examined by Western blotting. Vesicles derived from MDA-MB-231 cells were enriched with AHNAK. **d.** Protein from exosomes (Exo), microvesicles (MV), whole cell lysates (WCL), and whole cells lysates from MDA-MB-231 cells treated with 1 μM staurosporine for 24 hours (WCL+Stau) to induce apoptosis were examined by Western blotting for AHNAK, flotillin, TSG101, β-actin, Bax, and cleaved caspase 3 as indicated. Microvesicles contained AHNAK but not apoptotic markers (cleaved caspase-3 and BAX). Exosomes were enriched with Flotillin and TSG101, but not AHNAK nor apoptotic markers.

NTA analysis was used to examine the number and size of vesicles released into media by fibroblasts, MCF-7 cells, and MDA-MB-231 cells. As shown in Figure 3a, MDA-MB-231 cells produced the most extracellular vesicles, followed by less aggressive MCF-7 cells, and nontransformed fibroblasts. These data confirmed results obtained by electron microscopy. However, NTA analysis detected more vesicles smaller than 120 nm in diameter than seen by electron microscopy. This is consistent with the ability of NanoSight technology to detect smaller vesicles more efficiently than electron microscopy [17, 18].

### Extracellular vesicle composition defined by proteomic analysis and immunoblotting

Mass spectrometry (LC-MS/MS) identified over 270 proteins in extracellular vesicles produced by MDA-MB- 231 cells (see Supplementary Table S1). These data indicate that at over 1% of the entire proteome may be represented in extracellular vesicles from mammary carcinoma cells. While many of these proteins have been previously identified in extracellular vesicles, some may have not yet been reported. The most abundant vesicular proteins included AHNAK, annexin A2 (ANXA2), filamin A (FLNA), and three heat shock proteins including 78 kD glucose-related protein (HSPA5), HSP90 alpha (HSP90AA1), and HSP90 beta (HSP90B1) (Supplementary Table S1).

In particular, AHNAK was identified as the most prominent vesicle protein, along with several of its binding partners including annexin A2, annexin A6, myoferlin, and protein S-100 as shown in Figure 3b. As vesicles were mostly enriched with AHNAK, we sought to further validate this finding by immunoblot analysis. We confirmed that AHNAK is significantly expressed in extracellular vesicles as well as in the cell lines investigated as shown in Figure 3c.

Our next objective was to better characterize the vesicles isolated. Specifically, we wanted to address the possibility of contamination of extracellular vesicles with apoptotic bodies. We observed that these vesicles did not contain well known markers of apoptosis (i.e., BAX and cleaved caspase 3). In contrast, these apoptotic markers were found in positive controls (i.e. MDA-MB-231 staurosporine-treated lysate) as shown in Figure 3d.

We next determined whether exosomes also express AHNAK. Isolation of exosomes was successful, as demonstrated by enrichment with exosome markers (i.e. Flottilin-1, TSG-101). Interestingly, AHNAK was not detected in the exosomes, which points to microvesicles as the main source of AHNAK among the extracellular vesicles (Figure 3d).

### AHNAK localizes to cell membranes and vesicles

**Figure 4:**
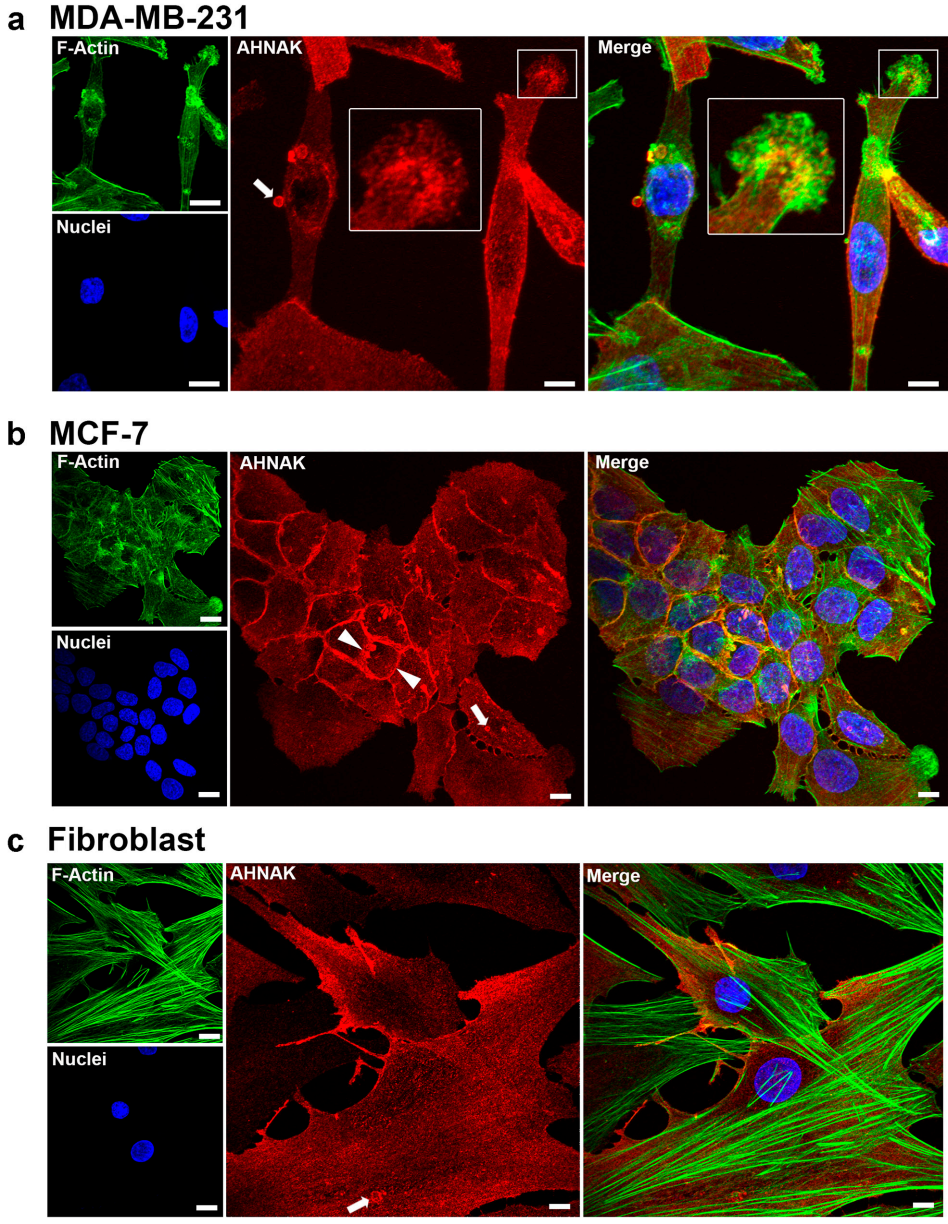
AHNAK localization in cell protrusions and extracellular vesicles AHNAK was visualized in cells by immunofluorescence confocal microscopy. **a.** MDA-MB-231 cells contained AHNAK at cell edges, as dot-like structures in the lamellipodia (insert), and in released vesicles (arrow). **b.** MCF-7 cells contained AHNAK mostly at intercellular junctions (arrowheads) and vesicles (arrows). **c.** Nontransformed fibroblasts contained AHNAK at cell edges and as small dots throughout the cytoplasm. Scale bars = 5 μm.

Confocal microscopy was used to examine the expression of AHNAK in mammary carcinoma cells and vesicles. As shown in Figure 4a, AHNAK was concentrated along the lateral plasma membrane, with more diffuse expression throughout the cytoplasm of MDA-MB-231 cells. AHNAK was also particularly concentrated at regions of intercellular contacts in MCF-7 cells (arrowhead in Figure 4b). In addition to these cell membrane and diffuse cytoplasmic locations, AHNAK showed punctate expression in vesicles (arrows in Figure 4a-4c).

**Figure 5:**
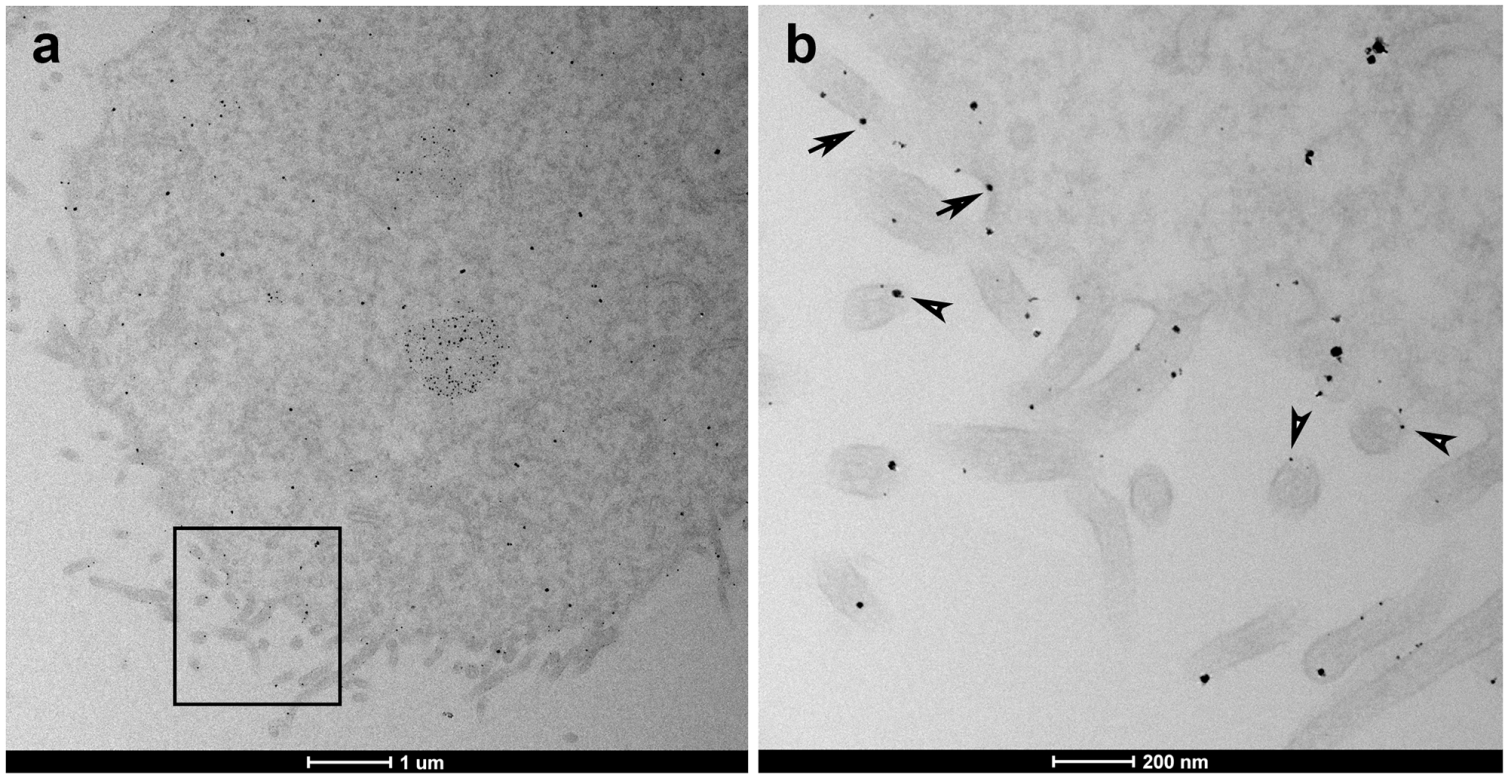
Subcellular localization of AHNAK in MDA-MB-231 cells **a.** TEM microscopy magnification shows general distribution of AHNAK. **b.** Higher magnification of the inset field finds that AHNAK was detected at vesicle-like structures (arrowheads) and near the plasma membrane or cell protrusions (arrows).

In order to further confirm the subcellular localization of AHNAK, we performed immunogold labeling of MDA-MB-231 cells and observed AHNAK presence at cell plasma membrane and vesicle-like structures (Figure 5). Our ultrastructural analysis revealed a staining pattern similar to that previously found for small vesicles termed enlargeosomes in neuronal cells [19].

**Figure 6:**
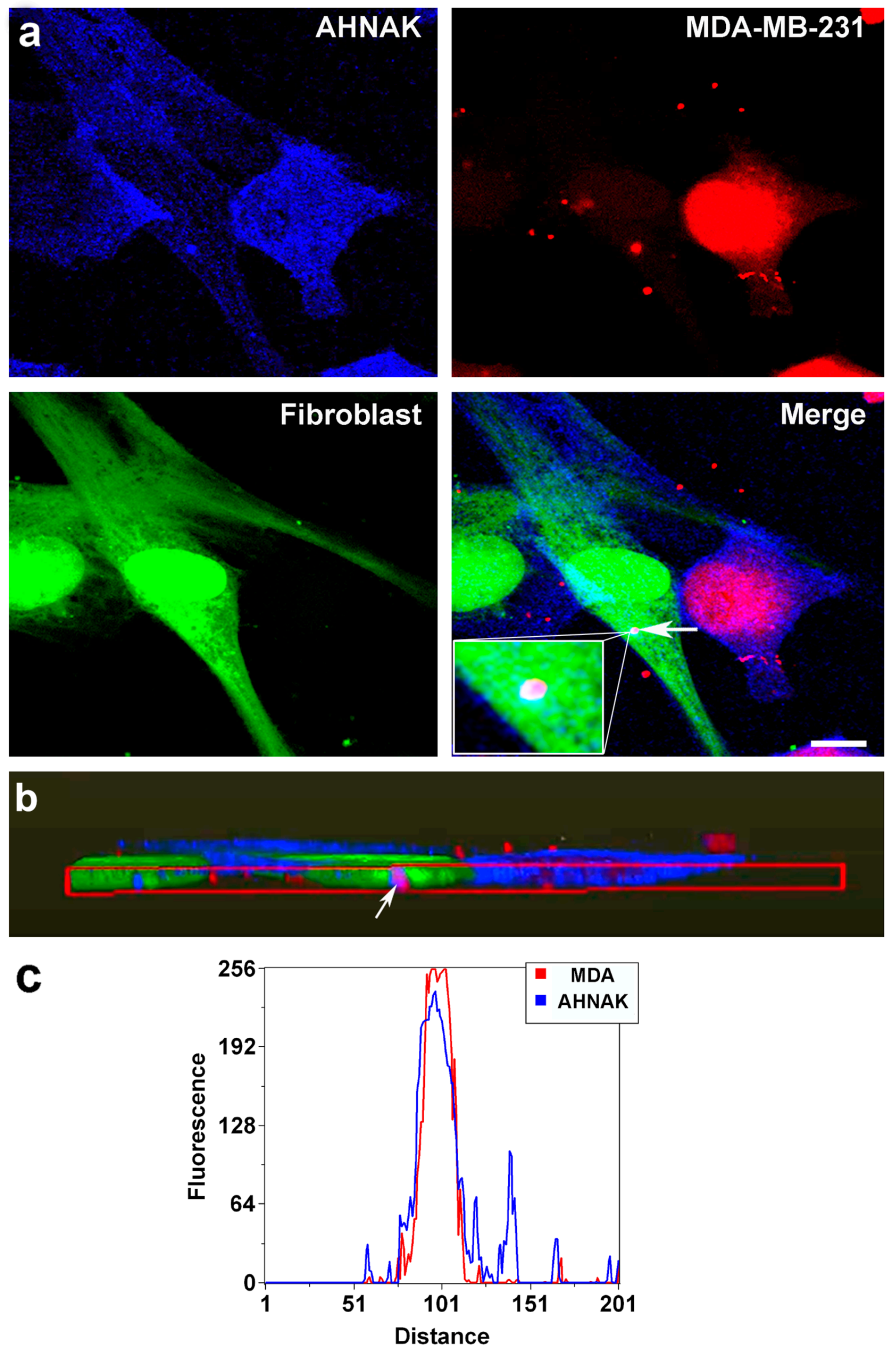
AHNAK localization in vesicles transferred from MDA-MB-231 cells to nontransformed fibroblasts **a.** MDA-MB-231 cells and fibroblasts were stained red and green, respectively, and cultured together for 48 hours before AHNAK was detected by immunofluorescence (blue). AHNAK can be seen in vesicles, including vesicles in fibroblasts that were produced by MDA-MB-231 cells (indicated by arrow). **b.** Volume rendering of confocal images to create an orthogonal plot showing a magenta vesicle derived from MDA-MB-231 cells and containing AHNAK inside a nontransformed fibroblast (indicated by arrow). Scale bar = 5 μm. **c.** Linescan profiles from the region indicate by arrow in (b) display a clear overlap between AHNAK (blue) and MDA-MB-231 vesicles (red).

Cells were co-cultured to examine AHNAK in vesicles that transferred from mammary carcinoma cells to neighboring fibroblasts. As shown in Figure 6a, AHNAK was present in vesicles produced by MDA-MB-231 cells that traveled to fibroblasts. This is particularly clear in merged images (arrow in Figure 6a) and orthogonal projections showing well defined vesicles inside fibroblasts as shown in Figure 6b. The graphic in Figure 6c shows an overlap between MDA-MB-231 vesicle (red channel) and AHNAK (blue channel). We, therefore, sought to investigate the role of AHNAK in mammary carcinoma extracellular vesicles.

### AHNAK is required for vesicle production and exchange

**Figure 7:**
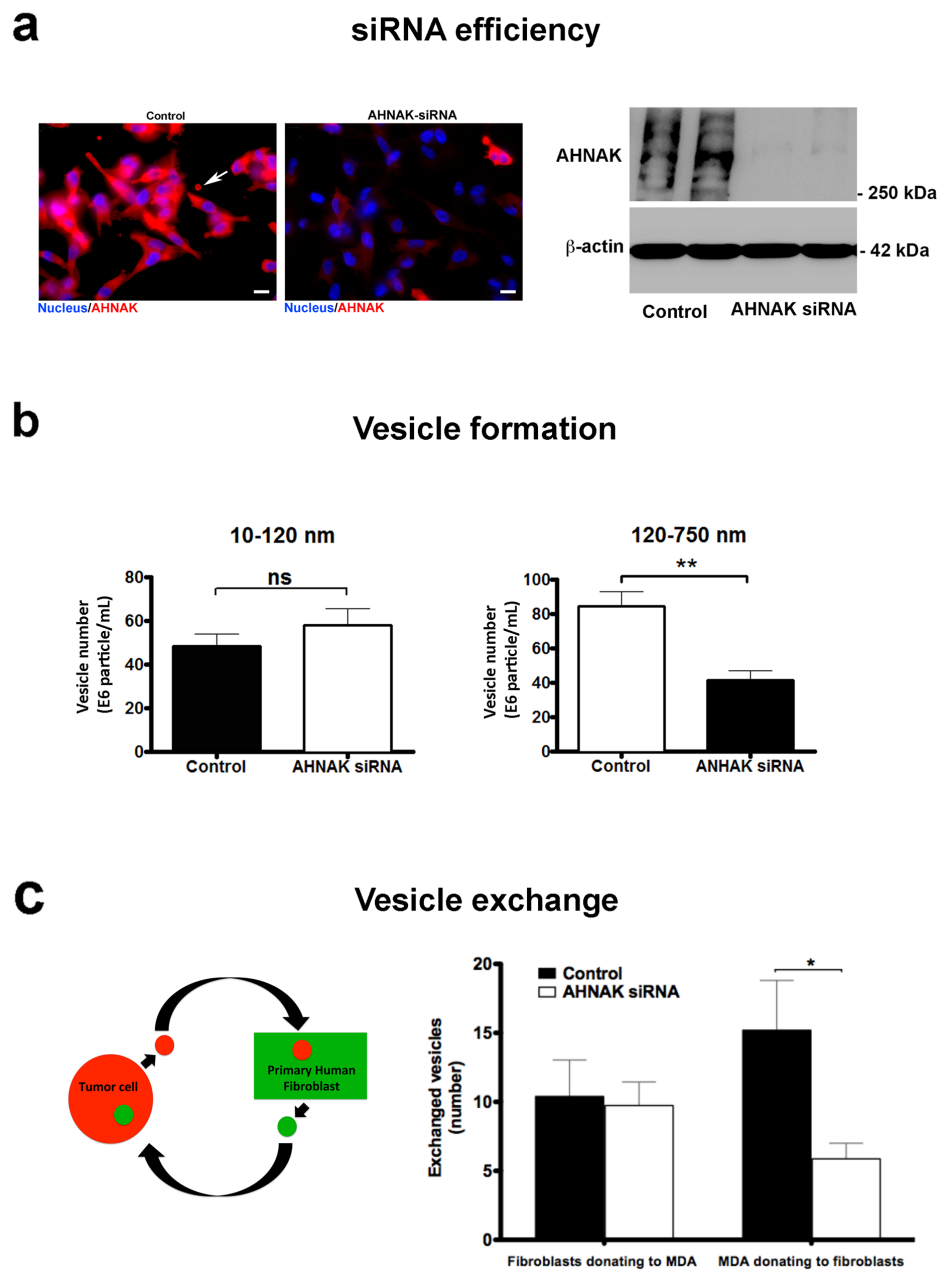
AHNAK depletion impairs microvesicle formation and exchange **a.** siRNA was used to deplete AHNAK expression in MDA-MB-231 cells, as observed by immunofluorescence (left panel) and immunoblot analysis (right panel). **b.** Cells with reduced AHNAK expression showed a significant decrease in microvesicle formation compared to controls. **c.** Depletion of AHNAK decreased vesicle transfer from MDA-MB-231 cells to fibroblasts. Data are shown as vesicles transferred between different cell types (mean+SEM, n=3). Single and double asterisks indicate statistically significant differences of p<0.05 and p<0.01, respectively.

The role of AHNAK in vesicle production and exchange was further explored by siRNA-mediated depletion of protein expression. Results from immunofluorescence microscopy and western blotting indicated that AHNAK production was efficiently targeted by this method in MDA-MB-231 cells (Figure 7a). We also found that AHNAK did not affect cell proliferation or viability of these cells (Supplementary Figure S1), indicating that AHNAK expression was not critical for mammary carcinoma cell growth.

These data were confirmed by nanoparticle tracking analysis. As shown in Figure 7b, AHNAK depletion decreased the number and size of vesicles present in conditioned medium of MDA-MB-231 cells compared to control cells. Interestingly, this effect seemed specific for larger vesicles. While the production of smaller vesicles with diameters of 10 to 120 nm (consistent with exosomes) was not significantly affected, AHNAK knockdown decreased the production of larger vesicles with diameters of 120 to 750 nm in diameter (consistent with microvesicles) by about 2 fold. Results from MDA-MB-231 cells cultured with nontransformed fibroblasts indicated that AHNAK knockdown caused a proportional decrease in the exchange of vesicles between these two cell types. As shown in Figure 7c, MDA-MB-231 cells transfected with AHNAK siRNA transferred about 2 fold less vesicles to fibroblasts than control cells.

**Figure 8:**
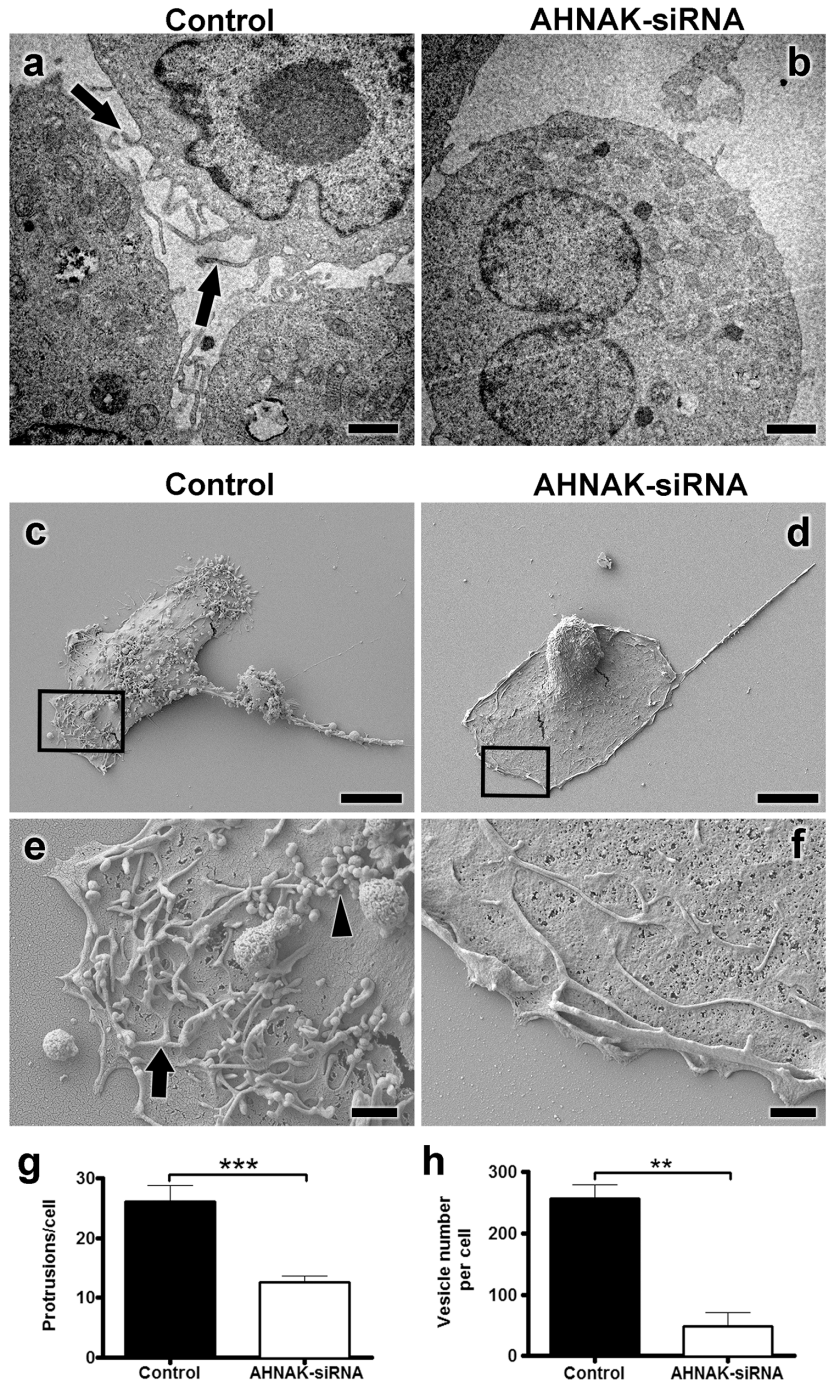
AHNAK downregulation abrogates formation of cell protrusions and extracellular vesicles MDA-MB-231 cells were transfected with scrambled or AHNAK siRNA and examined by electron microscopy as indicated. **a-b.** TEM analysis with cell protrusions indicated by arrows. **c-d.** SEM analysis of MDA-MB-231 cell surfaces. **e-f.** magnified regions outlined in panels C and D with examples of protrusions and vesicles indicated by arrows and arrowheads, respectively. Number of **g.** protrusions and **h.** vesicles per cell were quantitated and shown as protrusions or vesicles per cell (mean+SEM, n=20 in at least three different experiments). Double and triple asterisks indicate significant differences with p<0.01 and p<0.001 compared to control cells transfected with scrambled siRNA. Scale bars in A-B = 500 nm, C-D = 1 μm, and E-F = 10 μm.

The effects of AHNAK depletion on the formation of cell membrane protrusions and extracellular vesicles were also examined by electron microscopy. TEM analysis revealed that MDA-MB-231 cells transfected with AHNAK siRNA formed less cell protrusions than controls as shown in Figure 8a-8b. SEM analysis also found that AHNAK depletion decreased the number of vesicles produced by MDA-MB-231 cells compared to controls as shown in Figure 8c-8f. Quantitation of these data shown in Figure 8g-h indicates that AHNAK depletion significantly reduced the formation of protrusions and vesicles by over 2 fold and 5 fold, respectively. Results from electron microscopy indicated that AHNAK plays a functional role in the formation of cell protrusions and extracellular vesicles.

### AHNAK expression is increased in human breast tumors

**Figure 9:**
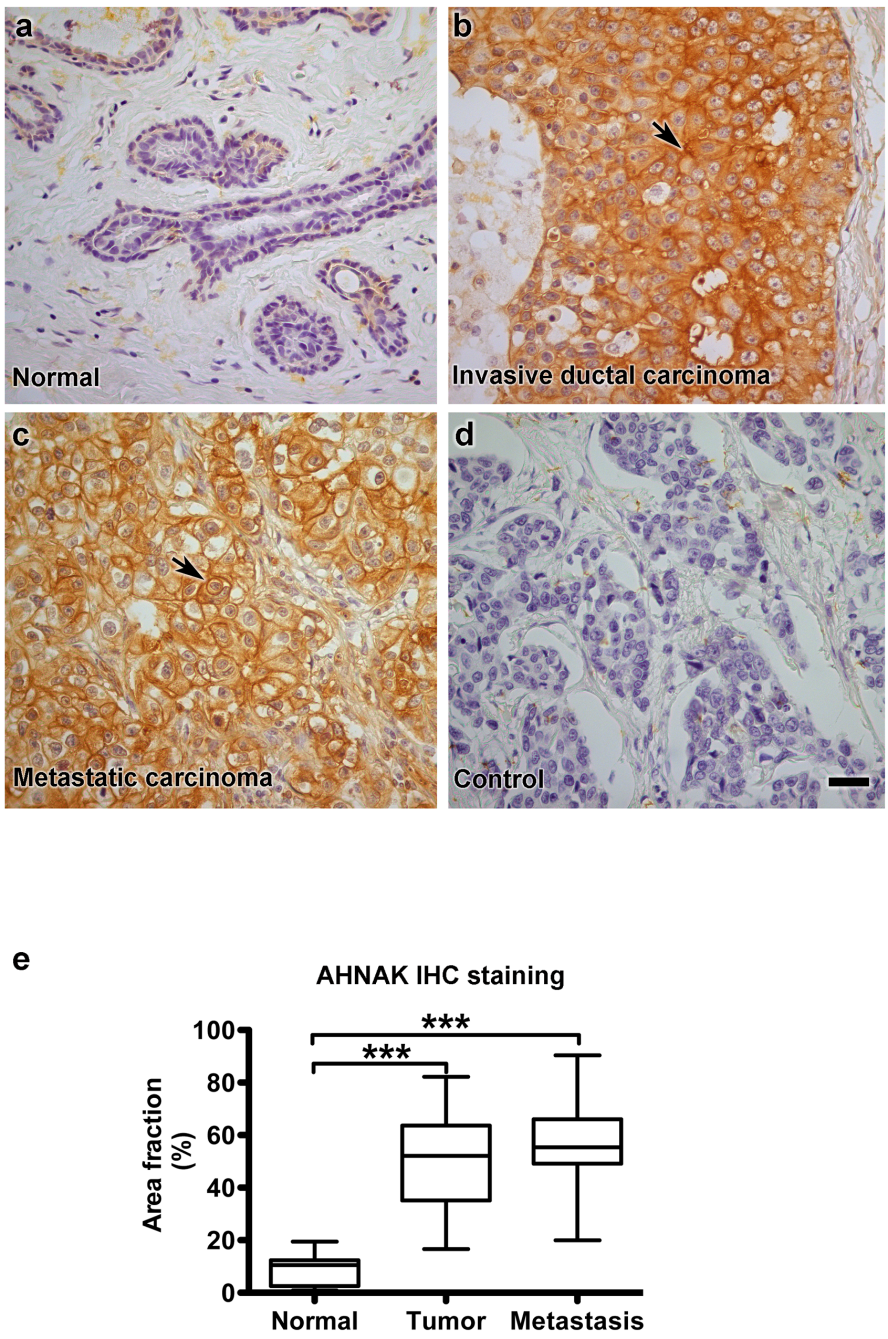
AHNAK is highly expressed in human mammary carcinoma cells *in vivo* AHNAK expression was evaluated in human clinical samples by immunohistochemistry. **a.** normal mammary epithelium, **b.** invasive ductal carcinoma, **c.** metastatic carcinoma from breast cancer patients, and **d.** invasive ductal carcinoma without primary antibody as a negative control. **e.** Quantitation of these data is shown as the percent of area with AHNAK expression (mean±SEM), and indicate that AHNAK expression was increased in invasive ductal carcinoma (n=35) and metastatic carcinoma (n=10) compared to normal tissue (n=9). Triple asterisks indicate significant differences compared to normal tissue with p<0.001. Scale bar = 20 μm.

Taken together, results from electron microscopy and nanoparticle tracking analysis suggest that AHNAK is needed for mammary carcinoma cells to produce microvesicles. Results from these *in vitro* experiments prompted us to compare the expression patterns of AHNAK in human clinical samples. AHNAK expression in normal mammary epithelium, invasive ductal carcinoma, and metastatic carcinoma were examined by immunohistochemistry as shown in Figure 9. Weak AHNAK staining was found in relatively few normal cells (Figure 9a). In contrast to normal cells, robust AHNAK expression was seen in the cytoplasm and plasma membrane of the majority of invasive ductal carcinoma cells (Figure 9b). Metastatic carcinoma cells contained the highest levels of AHNAK expression, particularly at the plasma membrane (Figure 9c). AHNAK staining seemed specific for carcinoma cells and was not prominent in stroma. Quantitation of these data indicates that AHNAK expression was significantly higher in mammary carcinoma cells than normal epithelium (Figure 9e).

### Extracellular vesicles from MDA-MB-231 cells promote fibroblast cell migration

**Figure 10:**
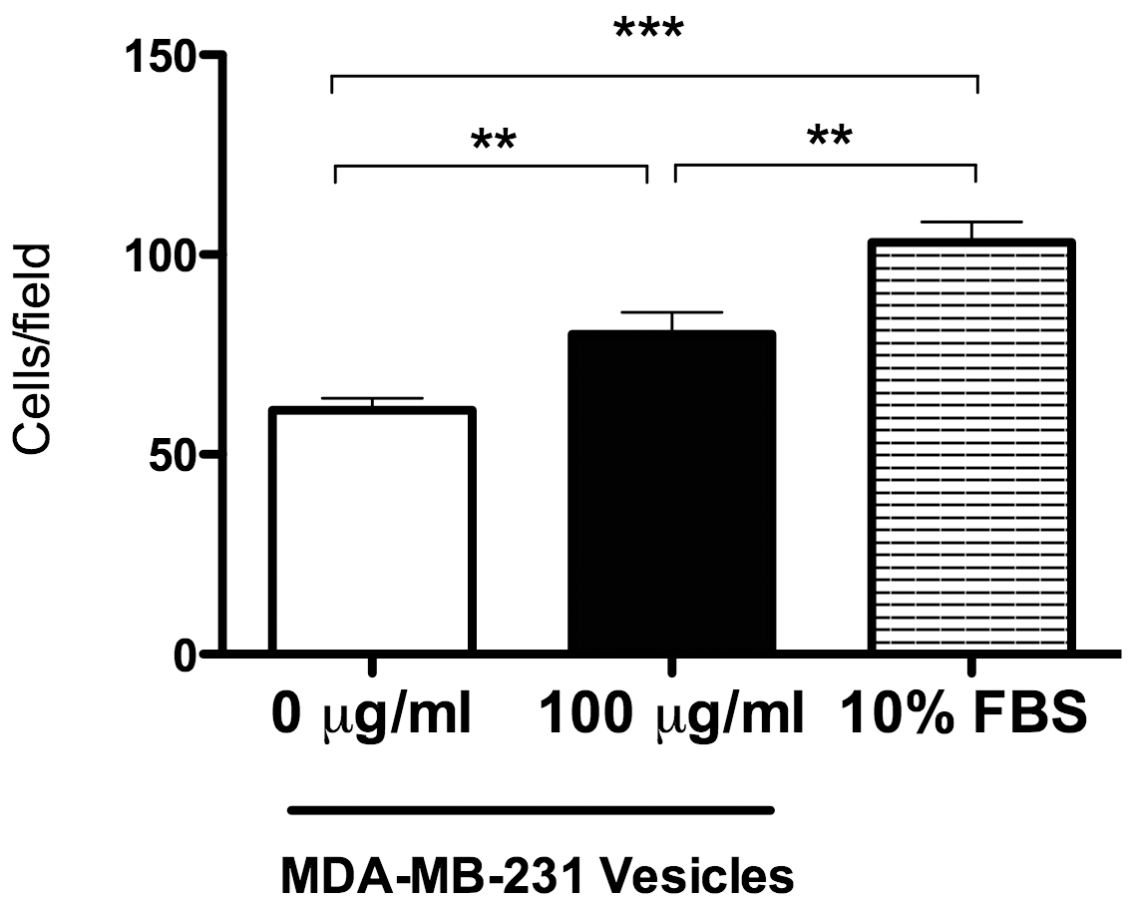
Extracellular vesicles derived from MDA-MB-231 cells promote fibroblast cell migration Nontransformed fibroblasts were plated on membrane with 8-micron pores in Transwell inserts with or without vesicles isolated from MDA-MB-231 cells as indicated, cultured in serum-free medium for 24 hours, and fixed with PFA. Cells on the upper side of the membranes were removed, and migrated cells on the lower side were stained with crystal violet, photographed, and counted. Cell motility was quantitated as the number of cells that migrated from the top of the membrane to the bottom of the membrane (mean+SEM, n=5). Double and triple asterisks indicate significant differences with p<0.01 and p<0.001, respectively, as indicated.

Results from imaging, mass spectroscopy, and siRNA experiments indicate that AHNAK supports the ability of mammary carcinoma cells to produce extracellular vesicles that enter neighboring fibroblasts. Fibroblasts can play important roles in promoting cancer progression in the tumor microenvironment [15, 20–22]. Increased migration is a hallmark of these cancer associated fibroblasts (CAFs). As shown in Figure 10a, nontransformed fibroblasts treated with vesicles from MDA-MB-231 cells migrated over 30% more than cells without vesicles. In contrast to cell migration, these vesicles did not increase fibroblast cell proliferation (data not shown). These data indicate that extracellular vesicles from mammary carcinoma cells can increase the motility of recipient mammary fibroblasts.

## DISCUSSION

Cancer development is driven by interactions between tumor cells and their surrounding stroma [6]. The tumor stroma is composed of an extracellular matrix and a variety of cells including fibroblasts, which can affect cancer progression [6, 23]. Emerging data indicate that critical interactions between tumor cells and their surrounding stroma are mediated by exchanges of extracellular vesicles [7]. In this study, we demonstrated the presence and exchange of vesicles between human mammary carcinoma cells and nontransformed fibroblasts from breast tissue. This communication was directional, with tumor cells producing more vesicles than the fibroblasts. Thus, net movement from tumor cells to fibroblasts may result from simple dynamics as opposed to molecular targeting.

Results from this study found vesicular structures ranging from 500 nm to 1 μm in diameter. Vesicles presenting size greater than 100 nm may originate by sprouting from the plasma membrane [8]. Thus, vesicles isolated and characterized in this study may be classified as microvesicles shed from the plasma membrane, as opposed to exosomes produced by exocytosis of multivesicular bodies and granules [7, 24, 25]. These data are consistent with production of microvesicles and exosomes by breast cancer cells reported by others [25, 26].

Our nonbiased analysis of extracellular vesicles produced by mammary carcinoma cells found AHNAK as the most prominent component. We confirmed the presence of AHNAK in isolated vesicles and cells by immunofluorescence and immunoblot analysis. AHNAK is a large protein of approximately 700 kDa found at the inner plasma membrane [27] in different cell types [28].

AHNAK participates in the formation of enlargeosomes, which are cytoplasmic vesicles first observed in neuronal cell lines [29]. Annexin-A2, which we found to be abundant in MDA-MB-231-derived vesicles, was also demonstrated to be present in enlargeosomes and to be necessary for its exocytosis [30]. Functionally, enlargeosomes can participate in processes including cell differentiation and membrane repair [29, 31]. Relatively low levels of AHNAK and myoferlin proteins have been found in vesicles produced by MCF-7 cells [13]. AHNAK has also been found in vesicles from primary human myotubes [32]. However, the functional relevance of AHNAK in mammary carcinoma derived vesicles has not been elucidated. Results from this study indicate that AHNAK is needed for vesicle production, and that these vesicles might augment tumor progression by mobilizing CAFs. These findings are consistent with reports of increased AHNAK expression in melanoma [33] and laryngeal cancer [34].

We found that depletion of AHNAK by siRNA did not affect mammary carcinoma cell growth in vitro, but inhibited the ability of these cells to produce microvesicles. This finding is consistent with the results of Shankar et al who demonstrated that AHNAK siRNA knockdown did not affect MDA-MB-231 cell viability, but resulted in pseudopod retraction, reduced actin cytoskeleton dynamics, reversion of EMT, and inhibition of cell migration and invasion [35]. Also, *AHNAK*-deficient mice are viable and present no developmental defects [36]. Taken together, our results indicate that AHNAK enables mammary carcinoma cells to form and release extracellular vesicles that travel into recipient fibroblasts.

It is becoming increasingly clear that CAFs remodel the tumor microenvironment to promote the invasion of neighboring carcinoma cells. This has been demonstrated in different types of squamous and glandular carcinomas [15, 20–22]. However, mechanisms that convert normal fibroblasts to tumor promoting CAFs have not been elucidated. Here, we found that mammary carcinoma cells produce vesicles that may play a role in this process.

Our data demonstrate that extracellular vesicles produced by tumor cells can increase motility of neighboring fibroblasts. However, concentrations of factors including serum, growth factors, and extracellular vesicles are likely to vary in the actual tumor microenvironment. Thus, the effects of extracellular vesicles on cell motility would be highly dependent on specific conditions in vivo. Nonetheless, pathways forged by these fibroblasts can be followed by carcinoma cells in the tumor microenvironment to augment tumor invasion and metastasis [20–22]. This scenario is supported by our finding that AHNAK production is elevated in invasive and metastatic human breast cancer cases. Thus, this process presents AHNAK, and potentially other components of extracellular vesicles, as new functionally relevant biomarkers and chemotherapeutic targets that can be used to detect and inhibit extracellular vesicles formation and tumorigenic intercellular communication.

## MATERIALS AND METHODS

### Cell lines and experimental culture conditions

MCF-7 and MDA-MB-231 mammary carcinoma cells were cultured in Dulbecco's modified Eagle's Medium-F12 (Sigma Chemical Co., St. Louis, MO, USA) supplemented with 10% fetal bovine serum (Gibco, Life Technologies, Eugene, Oregon, USA). Alternatively, MDA-MB-231 treated for 24h with 1μM Staurosporine served as a positive control for apoptosis. Nontransformed mammary fibroblasts were expanded from primary cultures derived from normal human breast tissue obtained from esthetic mammoplasty of a 22 year-old patient (Approved by Institutional Ethics Committee). These fibroblasts were maintained in DMEM (Sigma) supplemented with 10% FBS, 10 μg/ml ascorbic acid (Sigma), 5 U/ml penicillin solution and 5 μg/ml streptomycin (Gibco, Life Technologies). Cells were maintained in 75 cm^2^ flasks in a humidified atmosphere of 5% CO_2_ at 37°C.

### Co-culture

Nontransformed mammary fibroblasts were co-cultured with mammary carcinoma cells to analyze interactions between these cells. Briefly, fibroblasts were cultured over glass coverslips until reaching 90% confluence. Fibroblasts were loaded with green dye (Cell Tracker Green CMFDA, AM, Life Technologies). Mammary tumor cells, previously loaded with red dye (Cell Tracker Orange CMRA-AM, Life Technologies) were then plated over fibroblast monolayers. After tumor cell adhesion (24 hours), medium was removed and replaced with serum-free medium. After 24 hours, cells were fixed in 4% paraformaldehyde in phosphate-buffered saline (PBS) and coverslips were mounted with SlowFade Gold Antifade Reagent with DAPI (Life Technologies). Cells were analyzed with a Zeiss LSM 780 NLO confocal microscope (CEFAP-ICB), controlled by ZEN 2011 software. Special attention was devoted to interactions between the two lineages, represented by membrane events such as presence and exchange of vesicles.

### Isolation of extracellular vesicles from MDA-MB-231 cells

MDA-MB-231 cells (10^7^) were cultured in 150 cm^2^ flasks until 90% confluent, washed and cultured in serum-free medium for 24 hours. Conditioned media was removed, filtered through 0.8 μm pore membranes (Corning, NY, USA) to remove non-viable cells and debris, and centrifuged at 15.000 × g for 30 minutes at 4°C as previously described [37]. In order to further isolate exosomes, supernatant from the previous centrifugation was ultracentrifuged at 80,0000g for 3h at 4°C.

### Transmission electron microscopy (TEM) analysis

Cells were grown to 90% confluence before being washed and cultured in serum-free medium for 24 hours. Cells were then scraped off, pelleted, fixed in 2.5% glutaraldehyde in 0.1 M sodium cacodylate buffer (pH 7.2) overnight, and postfixed in 1% osmium tetroxide in the same buffer for 2 hours. Samples were then washed in distilled water, stained in bloc with 0.5% uranyl acetate, rinsed and dehydrated in graded ethanol. After immersion in propylene oxide, samples were embedded in epoxy resin (Spurr, Electron Microscopy Sciences, EMS, Hatfield PA, USA) and polymerized for 42 hours at 75°C. Sections (0.5 μm-thick) were stained with 1.0% toluidine blue in 1.0% aqueous sodium borate for light microscopic examination. Ultrathin sections were stained with lead citrate and uranyl acetate and examined in a JEOL 1010 transmission electron microscope (Jeol Inc., Peabody, MA, USA) at 80 kV.

Extracellular vesicles were obtained and pelleted by centrifugation as described above, suspended in PBS, and 5 μl of samples were deposited on carbon coated copper grids (CF100-Cu, EMS), incubated at room temperature overnight, and fixed with 2.5% glutaraldehyde for 5 minutes. Grids were washed with distilled water several times and stained with 1% uranyl acetate for 5 minutes, followed by TEM analysis.

### Scanning electron microscopy (SEM) analysis

Cells were plated and grown on coverslips for 24 hours, fixed with 2% glutaraldehyde in 0.1 M sodium cacodylate buffer (pH 7.2) for 2 hours, washed with PBS, and postfixed in 1% osmium tetroxide in the same buffer for 1 hour. The cells were then dehydrated in a series of increasing ethanol concentrations, dried in a critical point apparatus (Bal-Tec CPD-030 Critical Point Dryer, Bal Tec, Liechtenstein), and sputter-coated with gold (SCD-040, Bal Tec, Liechtenstein). Cells were examined in a JEOL JSM-7401F scanning electron microscope (SEM - JEOL JSM-7401F FESEM, Tokyo, Japan) at 5.0 kV.

### Extracellular vesicles analysis and quantification by nanoparticle tracking analysis (NTA)

Extracellular vesicles obtained from conditioned media were obtained as described above and suspended in sterile PBS. Extracellular vesicles number and size were counted with nanoparticle tracking analysis equipament (NanoSight LM10, Amesbury, UK), equipped with a charge-coupled device (CCD) camera and a laser emitting a 60-mW beam at 405 nm wavelength. Acquisitions were performed in five records of 60 seconds using the following parameters: shutter of 604, gain of 100, and threshold of 10. At least 1,000 particles were tracked in each sample. Brownian motion of particles was used to calculate particle size and concentration by NTA 3.0 software (NanoSight).

### Mass spectrometric analysis

Protein from extracellular vesicles isolated from MDA-MB-231 cells was subjected to tryptic digestion. Briefly, protein was incubated in 4M GuHCl and 5mM DTT at 65°C for 60 minutes. Iodoacetamide was then added to a final concentration of 15 mM and the samples were incubated for 60 minutes at room temperature. DTT was then added to a final concentration of 15 mM to quench excess Iodoacetamide. Digested protein was precipitated with 8 volumes of acetone and 1 volume methanol for 3 hours at −80°C, centrifuged 10.000 × *g* for 10 minutes, washed twice with methanol, and suspended in 2.5 mM NaOH followed by 50 mM HEPES buffer, pH 7.5 to a final volume of 100 μL. Trypsin (Proteomics grade; Sigma, St. Louis, MO, USA) was added at 1:100 ratio (enzyme/substrate), and protein samples were incubated at 37°C for 18 hours.

Tryptic peptides were desalted with Sep-Pak Vac C18 1cc (Waters, Milford, USA), vaccum dried, suspended in 10 μL of 0.1% formic acid. The peptide mixture was injected into a trap column (100 μm i.d. × 2 cm) packed with AQUA C18, 5 μm beads (Phenomenex), and then separated on a 10-cm long fused silica emitter packed with 1.9 μm-diameter Reprosil-Pur C-18-AQ beads. Nanoflow liquid chromatography was performed at a flow rate of 400 nL/min, on a Proxeon Easy nanoLC HPLC (Thermo Fisher Scientific, California, USA) coupled to an LTQ-Orbitrap Velos mass spectrometer (Thermo Fisher Scientific). Peptides were loaded onto the column with buffer A (0.1% acetic acid) and eluted with a 150 minutes gradient from 0 to 80% B (acetonitrile in 0.1% formic acid). The mass spectrometer was operated in data dependent mode, in which one full MS scan was acquired in the *m*/*z* range of 300-1650 followed by MS/MS acquisition using collision induced dissociation of the ten most intense ions from the MS scan. MS spectra were acquired in the Orbitrap analyzer at 30,000 resolution (at 400 *m/z*) and MS/MS scans were acquired in the linear ion trap. Dynamic exclusion was defined by a list size of 500 features and exclusion duration of 90 seconds. A target value of 1,000,000 was set for the survey (MS) scan, and the target value for the fragment ion (MS/MS) spectra was set to 40,000 ions. The lower threshold for targeting precursor ions in the MS scans was 5,000 counts.

Mass spectrometric (RAW) data were analyzed with MaxQuant software (version 1.5.0.1.2. A False Discovery Rate (FDR) of 1% was required for both protein and peptide identifications. The MS/MS spectra were searched against the UniProt database, restricted to *Homo sapiens* taxonomy. Enzyme specificity was set to trypsin and at least two missed cleavages were allowed; cysteine carbamidomethylation was selected as fixed modification whereas methionine oxidation and glutamine/asparagine deamidation were selected as variable modifications. Peptide identification was based on a search with an initial mass deviation of the precursor ion of 7 ppm and the fragment mass tolerance was set to 20 ppm.

### Depletion of AHNAK by siRNA

Cells were transfected with siRNA specific for AHNAK (Santa Cruz Biotechnology Inc., Santa Cruz, CA, USA), according to the manufacturer's instructions. Briefly, cells were incubated with a complex formed by the siRNA (10 μM), transfection reagent (Lipofectamine 2000, Life Technologies), and transfection medium (Opti-MEM I, Gibco, Life Technologies) for 48 hours at 37°C. Scrambled siRNA was used as a negative control. Cell viability of transfected cells was assessed by Trypan blue dye exclusion.

### Western blotting

Cells were lysed with RIPA buffer (150 mM NaCl, 1.0% NP-40, 0.5% deoxycholate, 0.1% SDS, 50 mM Tris pH 8.0) containing protease inhibitors (Sigma). After centrifugation (10,000 × g) for 10 minutes at 4°C, supernatants were recovered and quantified (BCA kit, Pierce Inc Rockford, IL, USA). Samples were suspended in Laemmli buffer containing 62.5 mM Tris–HCl (pH 6.8), 2% sodium dodecyl sulphate (SDS), 10% glycerol, 5% mercaptoethanol and 0.001% bromophenol blue. Equal amounts of protein (20 μg) from cell lysates and extracellular vesicles were electrophoresed on 6% polyacrylamide gels, transferred to Hybond ECL nitrocellulose membranes (Amersham), and blocked in Tris-buffered saline (TBS 1X) with 5% non-fat milk for 1 hour or TBS 1X with 0.05% Tween 20 (TBST), overnight at 4°C. Following one wash in TBST, membranes were probed with antibodies against AHNAK (1:1000, H-153, sc-98373, Santa Cruz Biotechnology) and β-actin (1:2000, Sigma), followed by appropriate secondary antibodies which were detected by chemiluminescence (ECL).

### Immunofluorescence

Cells were fixed with 4% PFA in PBS and permeabilized with 0.5% Triton X-100 (Sigma) in PBS for 5 minutes. Samples were then blocked with 10% normal goat serum (KPL, Gaithersburg, USA) for 1 hour, and incubated with primary antibody against AHNAK (mouse monoclonal clone E5; Santa Cruz Biotechnology Inc., Santa Cruz, CA, USA) diluted 1:50 in PBS for 1 hour at room temperature. Primary antibody was detected by goat anti-mouse secondary antibodies, conjugated to either Alexa Fluor 568 or Alexa Fluor 647 (Life Technologies, Eugene, Oregon, USA) for 1 hour protected from light. Samples were mounted in ProLong with DAPI to stain nuclei (Life Technologies). Non-immune serum served as negative controls.

Results were analyzed by fluorescence microscopy (Axiophot, Carl Zeiss, Oberkochen, Germany) using a PlanApo 100x objective (1.45NA). Cell images were acquired with a digital CCD monochromatic camera (CoolSnap HQ2, Photometrics Inc, Tucson, AZ, USA). The microscope and additional hardware were controlled by Metamorph Premier 7.6 software (Molecular Devices, Sunnyvale, CA, USA). Samples were also analyzed by confocal microscopy with a Leica TCS AOBS SP8 Tandem Scanner with spectral detection system Leica SP Detector (Leica Microsystems, Germany). Measurements of colocalization areas were determined using ImageJ public domain software (http://rsb.info.nih.gov/ij/). Colocalization analysis was carried out by the Linescan tool (Metamorph Premier 7.6 software), and the Image J plugin JaCop (http://rsb.info.nih.gov/ij/plugins/track/jacop.html).

### Immunogold electron microscopy

Cell pellets were fixed with 0.1% glutaraldehyde and 4% PFA in PBS for 1 hour. Fixative was removed by centrifugation and cells were washed twice in 0.05M glycine in PBS. After one wash in PBS, cells were permeabilized and blocked in 0.1% saponin and 5% goat serum for 30 min. Samples were then incubated with primary antibody against AHNAK (mouse monoclonal clone E5; Santa Cruz Biotechnology Inc., Santa Cruz, CA, USA) diluted 1:50 in 10% goat serum for 1 hour at room temperature. Primary antibody was detected by anti-mouse secondary antibody, conjugated to Nanogold 1.4nm (Nanogold, Yaphank, NY, USA) diluted 1:50 in 10% goat serum for 1 hour. Gold Enhance EM Plus was used according to manufacturer's instructions to enlarged gold particle size. Non-immune serum served as negative controls. Results were analyzed in a FEI Tecnai G20 Electron microscope at 200kVA.

### Cell proliferation assay

Cells were incubated for 24 hours with 60 μM BrdU (Sigma Chemical Co, St. Louis, MO, USA) in serum-free medium, fixed, and DNA was denatured in 2 N HCl for 30 minutes, then neutralized in 0.1 M borate buffer (0.1 M H_3_BO_3_, 0.15 M NaOH, pH 8.4) for 10 minutes. Cells were incubated with 0.3% Triton X-100 in PBS for 15 minutes and blocked with 1% bovine serum albumin (BSA, Sigma) and 0.1% Triton X-100 in PBS for 1 hour. Cells were then incubated with anti-BrdU biotinilated primary antibody (Biotin mouse, 1:100, MAB 3262B, Millipore) overnight, washed, and incubated with Alexa Fluor 555 streptavidin (1:500, Life Technologies, Eugene, Oregon, USA) secondary antibody for 1 hour. Cells were washed and nuclei stained with DAPI.

Images were taken from at least four fields by fluorescence microscopy (Axio vert A1, Carl Zeiss, Germany) using a LD-Plan 20x objective. Images were acquired with a digital AxioCam MRc camera (Carl Zeiss, Germany), and analyzed by ImageJ software to calculate the percentage of BrdU-positive nuclei from total nuclei number.

### Immunohistochemistry

Tissue microarray slides from normal human and breast cancer samples were obtained from Imgenex (San Diego, CA; IMH-364). Sections (4μm) from 54 samples were analyzed, including 35 cases of invasive ductal carcinoma (IDC), 10 cases of cancer metastasis, and 9 samples of normal breast tissue adjacent to cancer tissue.

Sections were deparaffinized in xylene and hydrated in decreasing ethanol concentrations. Antigen retrieval was carried out with citrate buffer (10 mM citric acid, 0.05% Tween 20, pH 6.0) in a water bath (95-100°C) for 30 minutes. Sections were blocked for 1 hour with 1% BSA (Sigma) in PBS. AHNAK was identified with a rabbit polyclonal antibody (1:100 in PBS, HPA026643, Sigma) overnight at 4°C. Endogenous peroxidase blocking was performed for 20 minutes, followed by Biotin-conjugated anti- rabbit secondary antibody (Dako) for 30 minutes. Diaminobenzidine (Sigma) was used as chromogen, and sections were counterstained with Mayer's hematoxylin (Sigma).

Brightfield images from five randomly selected fields from each sample were acquired with a Primostar microscope (Carl Zeiss, Germany) equipped with a CCD color camera (AxiocCam HRc, Carl Zeiss). All images were acquired at the same magnification (40x). Areas of diaminobenzidine staining were segmented using the color deconvolution plug-in of ImageJ (public domain software developed by Wayne Rasband, NIMH, NIH, Bethesda, MD, USA, http://rsbweb.nih.gov/ij/) to segment images and calculate the percent of AHNAK positive area.

### Cell migration assay

Migration assays were carried out in Transwell inserts with 8 μm pores in 12 well plates (Corning). Fibroblasts (5 × 10^4^) were plated into upper chambers in serum-free medium. Lower chamber were filled with vesicles derived from MDA-MB-231 cells (100μg/ml), diluted in serum-free medium. Complete media and serum-free media were also used as controls. Cells were cultured in these conditions for 24 hours before inserts were fixed with 4% PFA in PBS and post-fixed with 0.2% crystal violet in 20% methanol. Cells on the upper side of the filters were carefully removed with cotton swabs, and the migratory cells on the lower side of the filter were photographed on a Zeiss Axiovert A1 microscope and counted by ImageJ software. Five random fields were evaluated in each well.

### Statistical analysis

Data were analyzed with GraphPad Prism software v5.0 (GraphPad Software, Inc., San Diego, CA, USA). Student's t-test was performed to evaluate differences between two groups. Differences between three or more groups were assessed by analysis of variance (One-way or Two-way), followed by multiple comparisons tests. Differences were considered statistically significant when p ≤ 0.05.

## SUPPLEMENTARY MATERIALS FIGURES AND TABLES





## Abbreviations

CAFs: cancer associated fibroblasts
IHC: immunohistochemistry
NTA: nanoparticle tracking analysis
PFA: paraformaldehyde
SEM: scanning electron microscopy
TEM: Transmission electron microscopy.
