# Supplementary material for: AHNAK enables mammary carcinoma cells to produce extracellular vesicles that increase neighboring fibroblast cell motility
Source: Oncotarget. 2016 Jun 27;7(31):49998–50016. doi: 10.18632/oncotarget.10307 (PMC5226564; doi:10.18632/oncotarget.10307)
Supplement: Supplementary file 1 [file oncotarget-07-49998-s001.pdf]

AHNAK enables mammary carcinoma cells to produce extracellular vesicles that increase neighboring fibroblast cell motility

SUPPLEMENTARY FIGURE AND TABLE

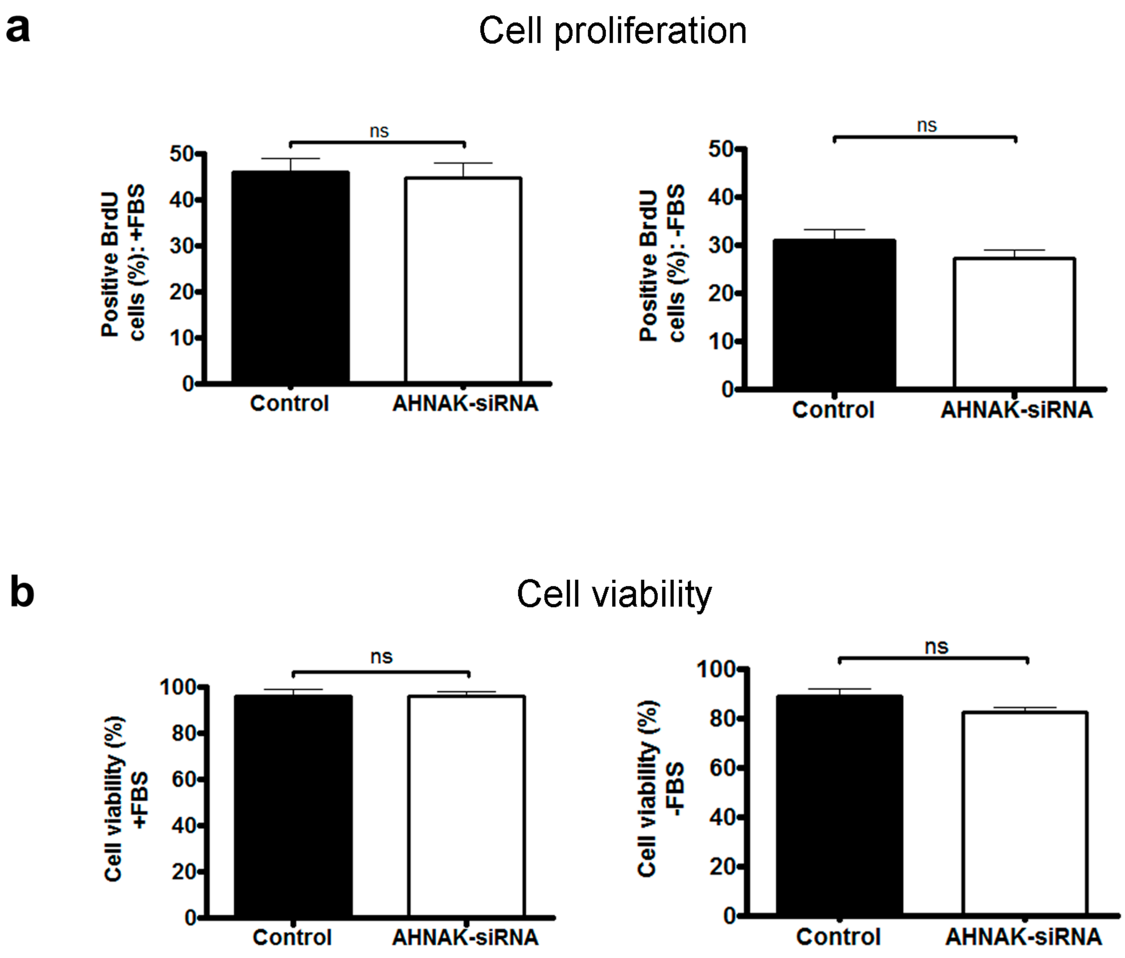

Supplementary Figure S1: AHNAK knockdown does not affect cell viability and proliferation of MDA-MB-231 cells. MDA-MB-231 cells were transfected with scrambled or AHNAK siRNA and cell proliferation and viability were evaluated by BrdU labeling and trypan blue dye exclusion, respectively. **a.** AHNAK knockdown does not affect the cell proliferation in the presence or absence of FBS. **b.** AHNAK knockdown does not affect the cell viability in the presence or absence of FBS.

Supplementary Table S1: Proteins identified in vesicles from MDA-MB-231 cells by proteomic analysis.

See Supplementary File 1
